# Author Correction: Spaceflight causes strain-dependent gene expression changes in the kidneys of mice

**DOI:** 10.1038/s41526-025-00467-y

**Published:** 2025-04-09

**Authors:** Rebecca H. Finch, Geraldine Vitry, Keith Siew, Stephen B. Walsh, Afshin Beheshti, Gary Hardiman, Willian A. da Silveira

**Affiliations:** 1 University of Staffordshire, Department of Sports and Science, School of Health, Education, Policing and Sciences, Science Centre, Leek Road, Stoke-on-Trent, ST4 2DF UK; 2 International Space University, 1 Rue Jean-Dominique Cassini, 67400 Illkirch-Graffenstaden, France; 3 Georgetown University Medical Center, Lombardi Comprehensive Cancer Center, Department of Oncology, 3970 Reservoir Rd, NW, New Research Building EP11, Washington, DC 20057 USA; 4 London Tubular Centre, Department of Renal Medicine, University College London, London, UK; 5 Center for Space Biomedicine, McGowan Institute for Regenerative Medicine, Department of Surgery, University of Pittsburgh, Pittsburgh, PA 15219 USA; 6 Stanley Center for Psychiatric Research, Broad Institute of MIT and Harvard, Cambridge, MA 02142 USA; 7 Faculty of Medicine, Health and Life Sciences, Institute for Global Food Security (IGFS), School of Biological Sciences, Queen’s University Belfast, 19 Chlorine Gardens, Belfast, BT9 5DL UK; 8 Department of Medicine, Medical University of South Carolina, MSC 403, 171 Ashley Ave Suite 419, Charleston, SC 29425 USA; 9 School of Science, Engineering and Environment. University of Salford, Manchester, M5 4WT UK

**Keywords:** Genetics, Systems biology, Computational biology and bioinformatics

Correction to: *npj Microgravity* 10.1038/s41526-025-00465-0, published online 25 March 2025

In this article the author name Afshin Beheshti was incorrectly written as Afshin Behesti. The original article has been corrected.

