# Supplementary material for: Spaceflight causes strain-dependent gene expression changes in the kidneys of mice
Source: NPJ Microgravity. 2025 Mar 25;11:11. doi: 10.1038/s41526-025-00465-0 (PMC11937539; doi:10.1038/s41526-025-00465-0)
Supplement: Supplementary file 1 — Supplementary Material [file 41526_2025_465_MOESM1_ESM.pdf]

Supplementary Data 1: C57BL/6J (RR-1 mission) differential gene expression analysis of mouse kidney spaceflight group compared to ground control group

Supplementary Data2: BALB/c (RR-3 mission) differential gene expression analysis of mouse kidney spaceflight group compared to ground control group

Supplementary Data 3: C57BL/6J (RR-1) functional enrichment analysis hallmarks of mouse kidney spaceflight group compared to ground control group

Supplementary Data 4: BALB/c (RR-3) functional enrichment analysis hallmarks of mouse kidney spaceflight group compared to ground control group

Supplementary Data 5: Genes with non-synonymous mutations between C57BL/6J and BALB/c lineages (Timmermans, Van Montagu and Libert, 2017)

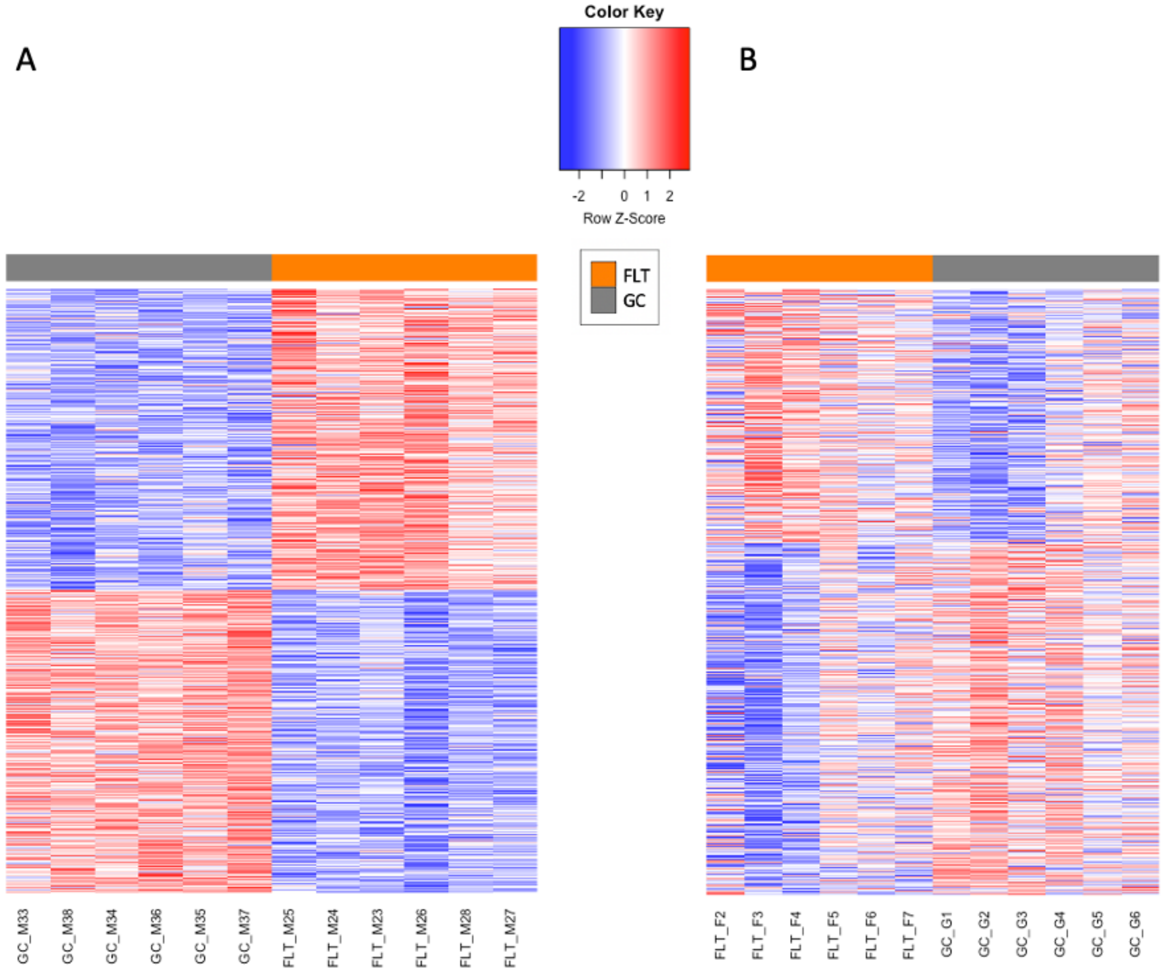

Supplementary Figure 1 Heatmaps of genes differentially expressed in spaceflight in kidney tissue from a) C57BL/6J (RR-1) and b) BALB/c (RR-3) (adjusted p-value <= 0.1). FLT = spaceflight group , GC = ground control group.

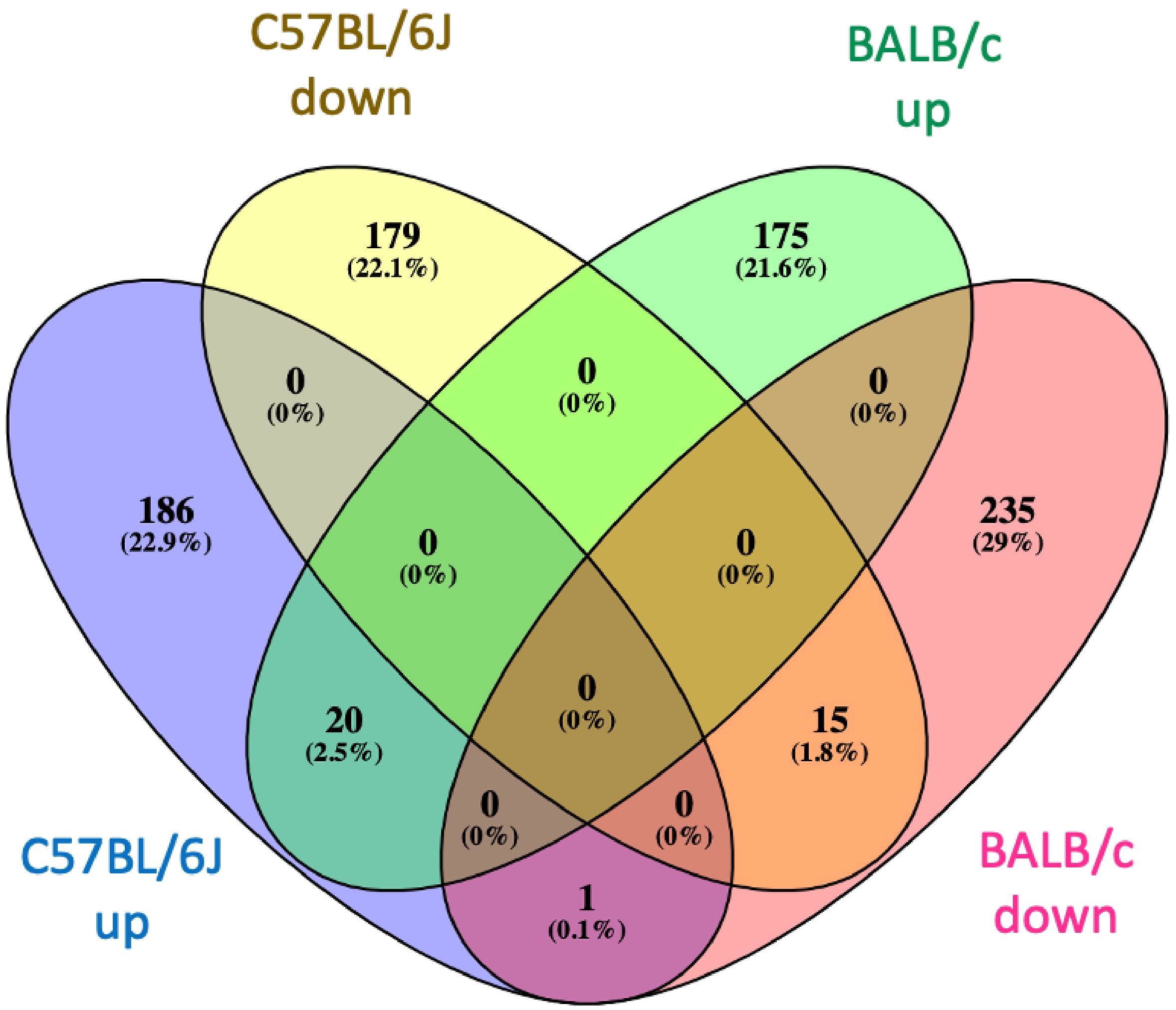

Supplementary Figure 2 Genes significantly upregulated and downregulated in differential expression determined by DESeq2 differential expression analysis on the effect of spaceflight on transcriptomic expression in C57BL/6J (RR-1) and BALB/c (RR-3) mice kidneys.

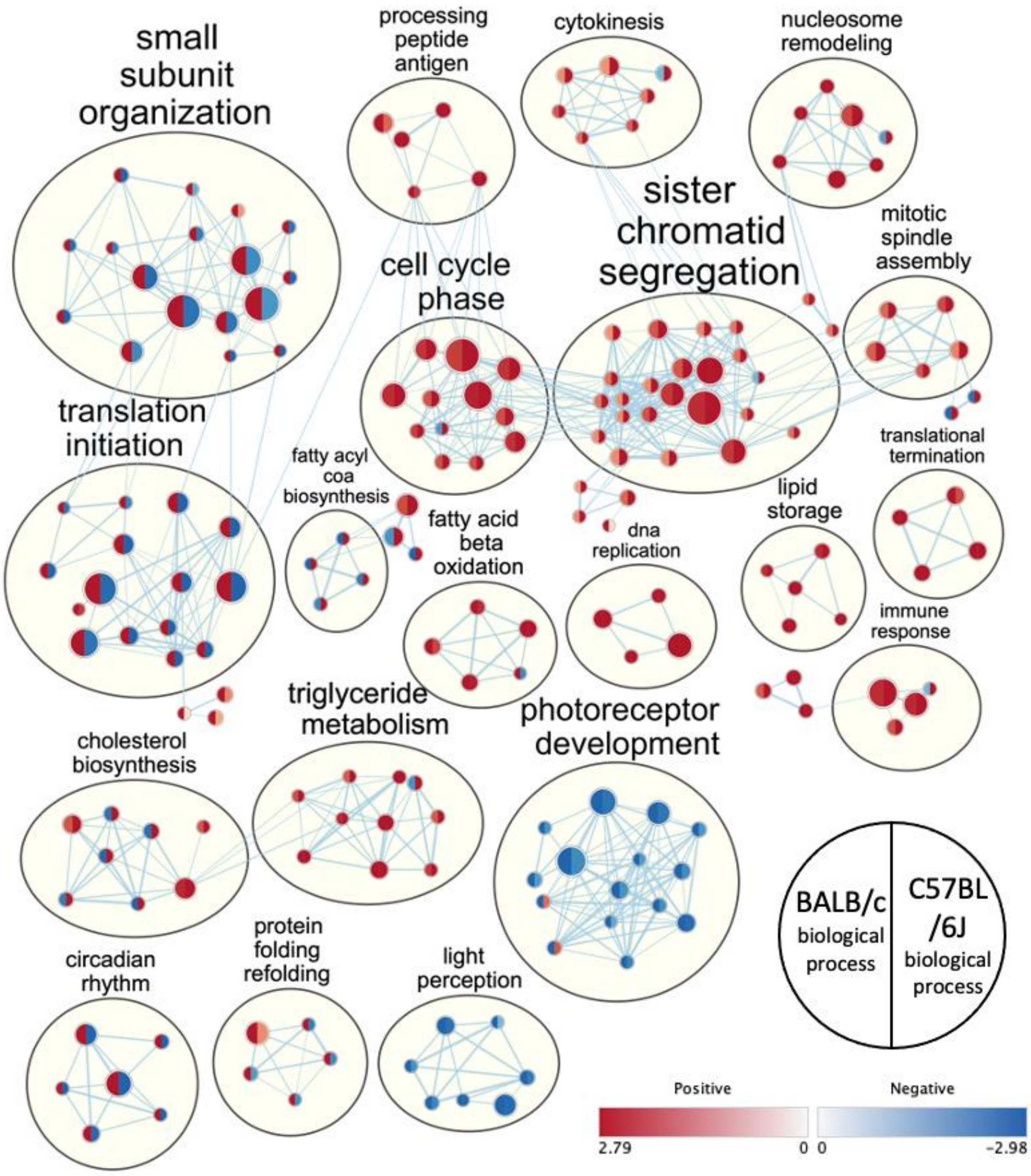

Supplementary Figure 3 Network analysis showing top 20 clusters in spaceflight in gene ontology biological processes between mouse kidney tissue data from C57BL/6J and BALB/c.

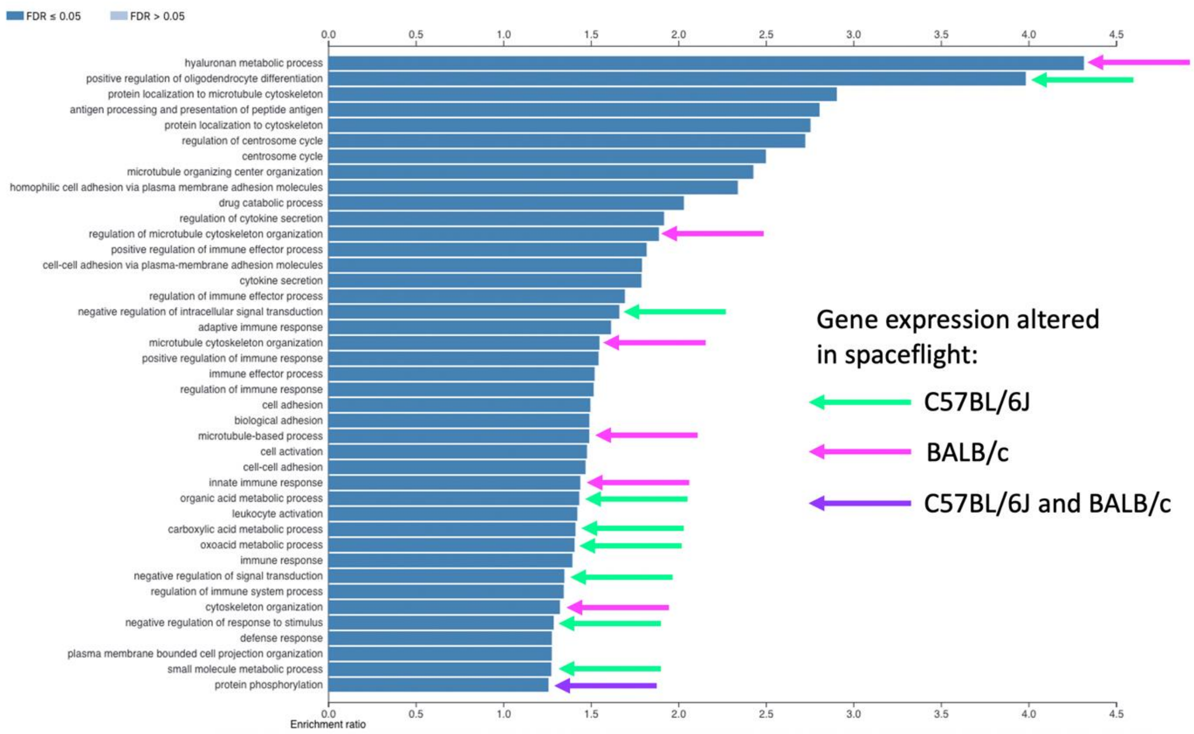

Supplementary Figure 4 Enriched biological process pathways of non-synonymous mutations in BALB/c mice compared to C57BL/6J strain reveal connections with expression of genes involved in pathways related to hyaluronan metabolism in BALB/c and intracellular signalling in C57BL/6J. Enriched biological process pathways in the genetics of non-synonymous mutations in BALB/c strain of mice compared to C57BL/6J strain (Timmermans, Van Montagu and Libert, 2017) and their connections with enriched pathways in the transcriptomic data of kidneys obtained from C57BL/6J mice (RR-1) and BALB/c (RR-3) in spaceflight.

Supplementary Table 1: Comparison of enriched biological processes from functional enrichment analysis of differentially expressed genes in mouse kidney spaceflight groups compared to ground control groups in C57BL/6J (RR-1) and BALB/c (RR-3) (FDR < 0.25)

	**RR1 up**	**RR1 down**
**RR3 up**	GOBP CENTROMERE COMPLEX ASSEMBLY<br>GOBP CHROMATIN REMODELING AT CENTROMERE<br>GOBP TRIGLYCERIDE METABOLIC PROCESS<br>GOBP BROWN FAT CELL DIFFERENTIATION<br>GOBP HISTONE EXCHANGE<br>GOBP TRIGLYCERIDE BIOSYNTHETIC PROCESS<br>GOBP DNA REPLICATION INDEPENDENT NUCLEOSOME ORGANIZATION<br>GOBP MITOCHONDRIAL TRANSLATIONAL TERMINATION<br>GOBP ANTIGEN PROCESSING AND PRESENTATION OF EXOGENOUS PEPTIDE ANTIGEN VIA MHC CLASS I<br>GOBP NEUTRAL LIPID BIOSYNTHETIC PROCESS<br>GOBP ANAPHASE PROMOTING COMPLEX DEPENDENT CATABOLIC PROCESS<br>GOBP POSITIVE REGULATION OF COLD INDUCED THERMOGENESIS<br>GOBP MITOCHONDRIAL TRANSLATION<br>GOBP CELL CYCLE DNA REPLICATION<br>GOBP DNA DEPENDENT DNA REPLICATION<br>GOBP TRANSLATIONAL TERMINATION<br>GOBP FOAM CELL DIFFERENTIATION<br>GOBP DNA REPLICATION INITIATION<br>GOBP REGULATION OF NUCLEASE ACTIVITY<br>GOBP PLASMINOGEN ACTIVATION<br>GOBP REGULATION OF MACROPHAGE DERIVED FOAM CELL DIFFERENTIATION<br>GOBP MITOCHONDRIAL GENE EXPRESSION	GOBP COTRANSLATIONAL PROTEIN TARGETING TO MEMBRANE<br>GOBP ESTABLISHMENT OF PROTEIN LOCALIZATION TO ENDOPLASMIC RETICULUM<br>GOBP NUCLEAR TRANSCRIBED MRNA CATABOLIC PROCESS NONSENSE MEDIATED DECAY<br>GOBP NUCLEAR TRANSCRIBED MRNA CATABOLIC PROCESS<br>GOBP PROTEIN LOCALIZATION TO ENDOPLASMIC RETICULUM<br>GOBP TRANSLATIONAL INITIATION<br>GOBP CIRCADIAN REGULATION OF GENE EXPRESSION<br>GOBP ENTRAINMENT OF CIRCADIAN CLOCK<br>GOBP POSITIVE REGULATION OF SIGNAL TRANSDUCTION BY P53 CLASS MEDIATOR<br>GOBP VIRAL GENE EXPRESSION<br>GOBP RRNA CATABOLIC PROCESS

	GOBP COENZYME A METABOLIC PROCESS<br>GOBP CELLULAR LIPID CATABOLIC PROCESS	
**RR3 down**	GOBP CALCIUM ION REGULATED EXOCYTOSIS OF NEUROTRANSMITTER<br><br>GOBP RESPONSE TO VITAMIN A<br>GOBP NEGATIVE REGULATION OF MICROTUBULE POLYMERIZATION OR DEPOLYMERIZATION	GOBP RENAL SYSTEM VASCULATURE DEVELOPMENT<br>GOBP RENAL TUBULE DEVELOPMENT<br><br>GOBP CARDIOCYTE DIFFERENTIATION
